# Poor sleep is associated with lower physical activity in a population-based cohort of middle-aged and older adults

**DOI:** 10.1038/s41598-025-10991-2

**Published:** 2025-07-17

**Authors:** Susanna C. Larsson, Emma Hållström, Karl Michaëlsson, Olga E. Titova

**Affiliations:** 1 Medical Epidemiology, Department of Surgical Sciences, Uppsala University, The EpiHub, Dag Hammarskjölds väg 14 B, Uppsala, 75185 Sweden; 2 Unit of Cardiovascular and Nutritional Epidemiology, Institute of Environmental Medicine, Karolinska Institutet, Stockholm, Sweden; 3 Department of Surgical Sciences, Uppsala University Hospital, Uppsala, Sweden

**Keywords:** Cohort, Sleep duration, Sleep disturbances, Sleep-disordered breathing, physical activity, Risk factors, Signs and symptoms

## Abstract

Sleep problems and inadequate physical activity (PA) are associated with numerous adverse health outcomes. Most studies explored the influence of PA on sleep, but how sleep affects engagement in PA across adulthood remains less well investigated. This study examined the association between sleep traits and PA reported the following year in 51,247 Swedish women and men (55–93 years), who completed questionnaires regarding their sleep, PA, and other characteristics. Health status was assessed with data from the Swedish National Patient Registry. Odds ratios and 95% confidence intervals were estimated by binary logistic regression. In multivariable analysis, long sleep duration (≥ 9 h/night), sleep disturbance, and symptoms of sleep-disordered breathing (SDB) were associated with lower odds of engaging in walking or cycling, and exercising the following year. In addition, short sleep duration (< 7 h/night), sleep disturbance, and symptoms of SDB were linked to sedentary behavior (time spent on reading or watching TV). Several sex- and age-specific associations were observed. In addition to previous evidence of a positive effect of PA on sleep, our findings indicate that poor sleep may contribute to lower engagement in PA and a higher level of sedentary behavior, highlighting the bidirectional nature of the relationship between PA and sleep.

**Supplementary Information:**

The online version contains supplementary material available at 10.1038/s41598-025-10991-2.

## Introduction

A large body of evidence indicates the beneficial effects of physical activity (PA) on health in healthy individuals and patient groups of all ages^1^. Regular PA has been associated with a reduced risk of multiple health outcomes, such as obesity, diabetes, cardiovascular diseases, cancer, cognitive impairment, psychiatric disorders, and mortality, as well as improved quality of life and well-being^1^. The World Health Organization defines PA as “any bodily movement produced by skeletal muscles that requires energy expenditure”^2^. This includes leisure-time PA (e.g., exercise, walking, gardening, and household work) or occupational PA as part of a person’s work or studying^2^. Clear recommendations for PA exist for different age groups. For adults, at least 150 min of moderate-intensity, or 75 min of vigorous-intensity PA, or an equivalent combination of moderate- and vigorous-intensity activity throughout the week is recommended^2,3^. A large proportion of the population does not meet these recommendations. Thus, the global age-standardized prevalence of insufficient PA is 28%, with a higher prevalence in high-income countries than in low-income countries^3^. Therefore, identifying factors that may affect engagement in PA is important.

Another emerging global health issue of concern is insufficient sleep and sleep-related disorders, which are increasingly common in modern societies and, similar to a lack of PA, have been associated with multiple disorders^4–9^. The relationship between PA and sleep can be bidirectional, as poor sleep may hamper efforts to be physically active. However, the majority of published studies have examined how PA affects sleep. Thus, experimental and observational studies suggested that PA has a beneficial effect on sleep, including improvement of sleep quality and sleep duration, shortened sleep latency, reduced sleep disturbances, and obstructive sleep apnea severity in both the general population and patients with sleep disorders^10–13^. These effects may be attributed to mechanisms such as reduced arousal and anxiety, improved circadian regulation, and increased energy consumption/metabolic rate following physical exertion. PA may also contribute to better sleep by improving mood, reducing stress, and promoting overall physical health^12^.

Conversely, poor sleep may contribute to lower engagement in PA, which could partly explain the previously observed associations between sleep disturbances and various health outcomes. Poor sleep may reduce motivation for PA by increasing fatigue, impairing cognitive and emotional functioning, and contributing to social withdrawal or unhealthy behaviors^14–18^. Moreover, sleep disturbances may impair physical recovery, disrupt appetite regulation, and lead to weight gain, all of which can further reduce PA levels^19,20^. Accordingly, PA may act as a mediator between sleep and health, in addition to having a direct health-promoting effect. However, the influence of sleep on subsequent engagement in PA, particularly among older adults in the general population rather than clinical or patient groups, has been less extensively studied. Previous observational studies have suggested that short sleep duration, insomnia symptoms, or poor sleep quality are associated with reduced PA in young adults and adolescents^21,22^, middle-aged or older participants^23–25^. Notably, previous studies had focused primarily on physical inactivity as the outcome, and typically examined only a single sleep-related exposure. In addition, several observational studies conducted in individuals with sleep disturbances, mental disorders, or other medical conditions have examined the association between sleep and PA, resulting in somewhat inconsistent findings^26^.

The present study examined the relationship between sleep patterns and future leisure-time PA. Specifically, we used a large cohort of 51,247 middle-aged and older Swedish women and men to longitudinally investigate the relationship between sleep duration, sleep disturbance, and symptoms of sleep-disordered breathing (SDB) measured at baseline with engagement in different PA and sedentary behaviours the following year. In addition, we sought to investigate if sex and age may modify these associations. We hypothesize that short and/or long sleep duration, as well as sleep disturbances, are associated with reduced engagement in PA the following year among middle-aged and older adults, and that these associations may be modified by sex and age.

## Methods

### Study population

The data from the National Research Infrastructure SIMPLER (Swedish Infrastructure for Medical Population-based Life-course Environmental Research) was used. Participants born between 1914 and 1952 were invited to participate in two longitudinal SIMPLER cohorts, the Swedish Mammography Cohort (SMC) and the Cohort of Swedish Men (COSM). Details of the study cohorts are shown in the **Supplementary Material** and have been previously reported^27^. Briefly, in 2008, subjects who participated in previous investigations and were still alive and resided in the study received a questionnaire about health, which included questions about sleep-related factors and other characteristics. In 2009, a Diet and Lifestyle Questionnaire that included detailed information about PA was sent to the participants. All participants who completed questionnaires in 2008 and 2009 were included in the main analysis (*n* = 51,247; 49% women), with a mean baseline age of 70 (age range of 56–95). The study was approved by the Swedish Ethical Review Authority (Dnr: 2019–03986). Informed consent has been obtained from the participants. The research was performed in accordance with the Declaration of Helsinki.

### Assessment of exposures, covariates, and outcomes

In 2008, participants completed questionnaires that solicited information about sleep duration, sleep disturbances, snoring, cessation of breathing, weight, height, employment status, cohabiting status, and history of depression.

Participants indicated how long they usually sleep and how often they experienced different sleep disturbance problems, sleep apnea or cessation of breathing, and disturbing snoring during the past 3 months with the following categories: never, seldom, often, mostly, and always.

Educational attainment was assessed in 1997. Health status was assessed with a Charlson weighted comorbidity index, defined based on the International Classification of Diseases, ICD codes (versions 8, 9, and 10) derived from the Swedish National Patient Registry^28^.

The questionnaire completed in 2009 included questions about levels of different PA during the past year. Our main outcomes of interest included PA during leisure time, which was ascertained with several multiple choice questions: time spent on *walking or bicycling* per day (hardly ever; <20 min/day; 20–40 min/day; 40–60 min/day; 60–90 min/day and > 90 min/day); *weekly leisure time exercise* (almost never, < 1 h/week; 1 h/week; 2–3 h/week; 4–5 h/week; >5 h/week); and time spent *watching TV or reading* (< 1 h/day; 1–2 h/day; 3–4 h/day; 5–6 h/day; 7–8 h/day; and > 8 h/day). Self-reported physical activity and inactivity have been previously validated against activity records and accelerometer data in a random subset of SMC participants^29^. The deattenuated concordance correlation coefficients comparing total daily PA versus accelerometer measurements and 7-day activity records were 0.38 and 0.64, respectively. Validity of leisure-time activity (walking/bicycling and exercise) and inactivity (watching TV/reading) estimates comparing the activity records with the questionnaire data were 0.42 and 0.52, respectively, suggesting reasonable validity^29^. PA and inactivity binary categories were created based on recommended minimum levels of daily PA for adults (met recommendations or not), i.e. 150–300 min of moderate-intensity, or 75–150 min of vigorous-intensity PA per week, or an equivalent combination of both^30^, and considering the structure of the questions and answer alternatives used in this study. In 2009, alcohol consumption and smoking status were also assessed.

### Statistical analysis

Descriptive data are presented as means (standard deviation, SD) or percentages. Binary logistic regression models were used to estimate odds ratios (ORs) and corresponding 95% confidence intervals (CIs) for specific PA variables. The main outcome variables were treated as binary variables: *walking or bicycling* (≤ 40 min/day *vs. >* 40 min/day); *leisure time exercise* (< 2 h/week vs. ≥ 2 h/week). In relation to *walking or bicycling*, the higher recommended value for moderate-intensity PA was chosen, i.e., 40 min/day, as approximately 76% of all participants reported walking or bicycling for at least 20 min per day. Since sedentary time was explicitly assessed as *watching TV or reading* and by 1–2 h category intervals, this variable was categorized as ≤ 4 h/day or > 4 h/day.

Main exposures, sleep characteristics were analysed separately in the main analysis. Habitual *sleep duration* was divided into 3 categories: short sleep, < 7 h per night; normal sleep, 7 to < 9 h of sleep; and long sleep, ≥ 9 h of sleep per night. *Sleep disturbances* were defined based on the following four symptoms: difficulty falling asleep, repeated awakenings with difficulty falling asleep, early awakenings, and disturbed sleep. Participants who reported that they suffered from at least one of the four above-mentioned sleep disturbance symptoms (often, most often, or always) were defined as having a sleep disturbance. Likewise, participants who reported that they experienced sleep apnea/cessation of breathing, or snoring often, mostly, or always were defined as having symptoms of SDB.

A basic model (Model A) included age (as continuous variable), sex, and education (less than high school, high school, or university). The Model B was further adjusted for employment status (working, or not working), cohabiting status (no/yes), cigarette smoking (never, former, current smokers), alcohol consumption (g/day), body mass index (BMI; calculated as weight divided by the square of height, kg/m^2^), health status (expressed as Charlson weighted comorbidity index)^31^, and history of depression (no/yes). Potential confounders were selected using directed acyclic graphs (DAGs)^32^ based on our knowledge of the relationships among potential confounders, intermediate variables, exposure, and outcome variables, as well as on existing information regarding factors associated with sleep and PA.

We examined multiplicative interactions of the experience of sleep-related problems with sex and age in relation to engagement in different PA in the fully adjusted Model B. Given that several sex-specific sleep-PA associations have been observed, the main analysis has been conducted in a total sample and split by sex. Although retirement age in Sweden is flexible, we used 65 years as a cut-off since it represented the average age of retirement^33^, and marks a common transition in daily routines and lifestyle among older adults. Among 51,247 participants, 2.4% had missing information in leisure time exercise, 1.8% on walking/bicycling habits, and 1% on watching TV/reading; information on sleep characteristics included in the analysis was missing between 1.7 and 7%. In addition, 7% had missing data on smoking, 3.6% on the employment status, 7% on education, 8.5% on cohabiting status, 3.2% on the history of diabetes, and 3.5% on weight or height (BMI). Missing information on these variables was imputed using multiple imputation with chained equations and 20 imputation cycles.

Several sensitivity analyses of the effect of sleep on PA were conducted. First, we restricted the study sample to apparently healthy individuals (Charlson Comorbidity Index = 0), as health disorders may greatly impact both PA and sleep. Given that PA before the baseline could affect both sleep and PA engagement, we adjusted for the respective PA measured in 1997 in the second sensitivity analysis. Due to a more significant number of missing values (15% for exercise, 14% for walking or bicycling, and 13% for sedentary behavior), imputations for these missing values have not been performed. Therefore, this sensitivity analysis was conducted on the available sample, including up to 44,752 individuals. All statistical tests were two-sided, and the analyses were performed using Stata version 15 (StataCorp, College Station, TX, USA).

## Results

### Sleep and physical activity in study participants

The baseline characteristics of participants are shown in Table 1. The majority (72%) were considered generally healthy, with a Charlson Comorbidity Index score of 0. One-third of the participants reported walking or bicycling for > 40 min daily, and 15% indicated leisure time exercise for ≥ 2 h per week. A high level of sedentary activities, defined as > 4 h per day reading or watching TV, was reported by 7.5% of participants. Habitual short sleep duration (defined as < 7 h/night) was reported by 32% of participants, and 7% met the criteria for long sleep duration (defined as ≥ 9 h/night); the proportion of those who reported short or long sleep was higher in women than men. In total, 43% had at least one of the four sleep disturbance symptoms (occurring often, most often, or always) and were defined as having a sleep disturbance. Among them, early awakenings were the most prevalent sleep disturbance complaint (30%), followed by repeated awakenings with difficulty falling asleep (28%), disturbed sleep (25%), and difficulty falling asleep (20%) in a total sample. All four sleep complaints were more prevalent in women than men. Moreover, 24% of participants reported having at least one SDB symptom (disturbed snoring or cessation of breathing). Both sleep apnea/cessation of breathing and snoring were more often reported by men than women (Table 1).

**Table 1:** Characteristics of the study participants.

Characteristics*	Total	Women	Men
**Number of participants**, n (%)	51,247	25,241 (49.3)	26,006 (50.7)
**Age**, years	70.0 (8.1)	70.5 (7.7)	69.5 (8.4)
**Education**, n (%)			
*≤ 9 years*	15,279 (32.1)	7,548 (34.7)	7,731 (29.8)
*10–12 years*	22,255 (46.7)	9,063 (41.7)	13,192 (50.8)
*> 12 years*	10,144 (21.3)	5,118 (23.6)	5,026 (19.4)
**Full-time or part-time employed**, n (%)	13,455 (27.2)	5,458 (22.3)	7,997 (32.1)
**Cohabiting**, n (%)	34,679 (74.0)	13,433 (59.7)	21,246 (87.2)
**Cigarette smoking**, n (%)			
*Non-smokers*	24,422 (51.4)	13,372 (57.2)	11,050 (45.8)
*Former smokers*	18,530 (39.0)	7,565 (32.3)	10,965 (45.5)
*Current smokers*	4,551 (9.6)	2,459 (10.5)	2,092 (8.7)
**Alcohol intake**, g/day, *median (Q1-Q3)*	4.3 (0.9–10.5)	2.5 (0.5–6.9)	6.8 (2.1–13.8)
**Body mass index**, kg/m^2^	26.0 (4.1)	25.7 (4.5)	26.3 (3.6)
**Charlson comorbidity index**, n (%)			
*0*	36,875 (72.0)	18,450 (73.1)	18,425 (70.9)
*1*	6,947 (13.6)	2,984 (11.8)	3,963 (15.2)
*≥ 2*	7,425 (14.5)	3,807 (15.1)	3,618 (13.9)
**History of depression**, n (%)	4,479 (8.7)	2,915 (11.6)	1,564 (6.0)
**Walking/bicycling > 40 min/day**, n (%)	16,962 (33.7)	8,191 (33.2)	8,771 (34.2)
**Exercise ≥ 2 h/week**, n (%)	7,580 (15.2)	3,735 (15.3)	3,845 (15.1)
**Reading/watching TV > 4 h/day**, n (%)	3,826 (7.5)	1,975 (7.9)	1,851 (7.2)
**Habitual sleep duration**, hours/night, n (%)			
*< 7*	16,040 (31.9)	8,318 (33.8)	7,722 (30.0)
*7 - <9*	30,889 (61.3)	14,405 (58.5)	16,484 (64.1)
*≥ 9*	3,426 (6.8)	1,922 (7.8)	1,504 (5.9)
**Difficulty falling asleep**, n (%)	9,991 (20.1)	6,841 (28.4)	3,150 (12.3)
**Repeated awakenings with difficulty falling asleep**, n (%)	13,693 (28.2)	8,417 (35.9)	5,276 (21.1)
**Early awakenings**, n (%)	14,692 (30.4)	8,112 (34.7)	6,580 (26.4)
**Disturbed sleep**, n (%)	11,920 (24.9)	6,929 (30.4)	4,991 (19.9)
**Sleep apnea/cessation of breathing**, n (%)	3,671 (7.7)	1,024 (4.5)	2,647 (10.8)
**Disturbing snoring**, n (%)	11,391 (23.5)	3,958 (16.9)	7,433 (29.6)

*Values are means ± SD or percentages. Some characteristics have a total number less than 51,247 due to missing values that were imputed prior to the statistical analysis.

### Sleep characteristics and engagement in PA and sedentary behavior the following year

In the fully adjusted model B, in a total sample, long sleep duration, sleep disturbance, and symptoms of SDB were associated with lower odds of walking or cycling and exercise the following year (Table 2). Short sleep duration was linked to lower odds of walking/cycling and exercise in the model adjusted for age, sex, and education (OR [95% CI] = 0.93 [0.89, 0.97] and 0.94 [0.89, 0.99], respectively). However, these relationships were attenuated after adjustment for other confounders (Table 2). There was a statistically significant interaction of sleep disturbance and symptoms of SDB with sex in relation to walking/cycling. A somewhat stronger inverse association between sleep disturbance and walking/cycling was found in women than men, whereas the link between symptoms of SDB and walking/cycling was more pronounced in men (Table 2).

**Table 2:** Associations between sleep variables and engagement in leisure-time physical activity and sedentary behavior, *n* = 51,247.

Sleep characteristics (exposures)	Total		Women (*n* = 25,241)	Men (*n* = 26,006)	
	**Model A** ^*^	**Model B** ^**^	**Model B** ^**^	**Model B** ^**^	**P-value** for sex-sleep interaction
	OR (95% CI)	OR ((95% CI)	OR ((95% CI)	OR ((95% CI)	
	**Walking/cycling > 40 min/day**				
**Sleep duration** (h/night)					
*< 7*	**0.93 (0.89–0.97)**	0.97 (0.93–1.01)	0.95 (0.90–1.01)	1.01 (0.95–1.07)	0.057
*7 - <9*	Ref	Ref	Ref	Ref	
*≥ 9*	**0.78 (0.72–0.84)**	**0.79 (0.73–0.85)**	**0.81 (0.73–0.91)**	**0.75 (0.67–0.85)**	0.994
**Sleep disturbance** ^**#**^					
No	Ref	Ref	Ref	Ref	
Yes	**0.85 (0.82–0.88)**	**0.86 (0.83–0.90)**	**0.83 (0.79–0.88)**	**0.90 (0.85–0.95)**	**0.021**
**Symptoms of SDB** ^**#**^					
No	Ref	Ref	Ref	Ref	
Yes	**0.75 (0.72–0.79)**	**0.83 (0.79–0.87)**	**0.87 (0.80–0.94)**	**0.81 (0.76–0.86)**	**0.038**
	**Exercise ≥ 2 h/week**				
**Sleep duration** (h/night)					
*< 7*	**0.94 (0.89–0.99)**	0.98 (0.93–1.03)	0.99 (0.92–1.07)	1.02 (0.94–1.10)	0.059 **§**
*7 - <9*	Ref	Ref	Ref	Ref	
*≥ 9*	**0.80 (0.71–0.89)**	**0.81 (0.72–0.90)**	0.93 (0.80–1.08)	**0.70 (0.59–0.84)**	0.101 **§**
**Sleep disturbance** ^**#**^					
No	Ref	Ref	Ref	Ref	
Yes	**0.92 (0.87–0.97)**	**0.92 (0.87–0.97)**	**0.90 (0.84–0.97)**	0.94 (0.87–1.01)	0.291
**Symptoms of SDB** ^**#**^					
No	Ref	Ref	Ref	Ref	
Yes	**0.87 (0.82–0.93)**	**0.93 (0.87–0.99)**	0.99 (0.90–1.09)	**0.89 (0.83–0.97)**	0.063
	**Reading/watching TV > 4 h/day**				
**Sleep duration** (h/night)					
*< 7*	**1.23 (1.15–1.33)**	**1.21 (1.12–1.30)**	**1.12 (1.02–1.24)**	**1.30 (1.17–1.45)**	0.057
*7 - <9*	Ref	Ref	Ref	Ref	
*≥ 9*	1.12 (0.98–1.27)	0.96 (0.84–1.09)	0.86 (0.72–1.03)	1.08 (0.89–1.31)	0.089
**Sleep disturbance** ^**#**^					
No	Ref	Ref	Ref	Ref	
Yes	**1.31 (1.23–1.41)**	**1.22 (1.14–1.31)**	**1.20 (1.09–1.33)**	**1.23 (1.11–1.36)**	0.733
**Symptoms of SDB** ^**#**^					
No	Ref	Ref	Ref	Ref	
Yes	**1.25 (1.15–1.35)**	**1.14 (1.05–1.23)**	1.04 (0.92–1.18)	**1.21 (1.09–1.35)**	**0.046**

CI, confidence interval; OR, odds ratio; Ref, reference group for the analysis. *Model A was adjusted for age, sex, and education based on binary logistic regression. **Model B was further adjusted for employment status, cohabiting status, cigarette smoking, alcohol consumption, body mass index, BMI, Charlson Comorbidity Index, and history of depression. # Participants reported that at least one symptom (see methods for description) occurred often, most often, or always. Bold values = P-values < 0.05. § P-value for interaction of sleep duration as continuous variable by sex was < 0.05.

In contrast, in the main analysis, short sleep duration, sleep disturbance, and symptoms of SDB were associated with higher odds of sedentary activities, i.e., reading or watching TV (Table 2). A statistically significant interaction of symptoms of SDB with sex was found in relation to reading or watching TV. Subsequent multivariable analyses revealed an association between symptoms of SDB and reading or watching TV in men but not in women (Table 2).

A statistically significant interaction of symptoms of SDB with age was observed in relation to exercise (P for interaction = 0.001, fully adjusted model B). Age-specific fully adjusted analyses revealed a significant inverse association between symptoms of SDB and exercise in middle-aged participants (< 65 years of age) but not in older participants (≥ 65 years of age; **Model B**, Table 3). Moreover, an interaction of sleep duration with age was observed in relation to variable reading/watching TV (P for interaction = 0.001, fully adjusted model B). The link between short sleep duration and higher odds of reading/watching TV > 4 h per day was more pronounced in middle-aged participants than in older individuals (Table 3).

**Table 3:** Age specific associations between several sleep variables and engagement in leisure-time physical activity and sedentary behavior, *n* = 51,247.

	Age < 65 years (*n* = 17,053)	Age ≥ 65 years (*n* = 34,194)
	OR (95% CI)	OR ((95% CI)
**Exercise ≥ 2 h/week**		
**Symptoms of SDB** ^**#**^		
No	Ref	Ref
Yes	**0.86 (0.78–0.95)**	0.97 (0.90–1.05)
**Reading/watching TV > 4 h/day**		
**Sleep duration** (h/night)		
*< 7*	**1.42 (1.20–1.68)**	**1.15 (1.06–1.25)**
*7 - <9*	Ref	Ref
*≥ 9*	1.20 (0.88–1.65)	0.91 (0.79–1.05)

CI, confidence interval; OR, odds ratio; Ref, reference group for the analysis.

Adjusted for exact age, sex, education, employment status, cohabiting status, cigarette smoking, alcohol consumption, body mass index, BMI, Charlson Comorbidity Index, and history of depression.

#Participants reported that at least one symptom (see methods for description) occurred often, most often, or always. Bold values = P-values < 0.05.

### Sensitivity analyses

A sensitivity analysis restricted to apparently healthy participants (no co-morbidities, defined as Charlson Comorbidity Index = 0; *n* = 36,875, 50% women), revealed results similar to those presented in Table 2 (Supplementary Table 1). But, the association between sleep disturbance and exercise was attenuated (OR [95% CI] = 0.95 [0.90, 1.00], *p* = 0.102, fully adjusted model). In a second sensitivity analysis, the findings for PA outcomes were similar after additional adjustment for the respective PA measured approximately 12 years earlier, in 1997 (Supplementary Table 2). However the relationship between symptoms of SDB and exercise was attenuated in the fully adjusted model (OR [95% CI] = 0.95 [0.89, 1.02], *p* = 0.177).

## Discussion

The present large population-based study of 51,247 middle-aged and older individuals revealed that a significant proportion of participants had short sleep (32%) or long sleep duration (7%), exhibited sleep disturbances (43%), or showed symptoms of SDB (24%). Our findings suggest that long sleep duration, sleep disturbance, and symptoms of SDB were associated with lower odds of engaging in PA, walking or cycling, and exercising the following year. In addition, short sleep duration, sleep disturbance, and symptoms of SDB were linked to sedentary behavior, such as time spent reading or watching TV. Several sex- and age-specific associations were observed. Given that general health and PA levels before baseline may influence sleep exposures and PA outcomes, several sensitivity analyses were conducted. Similar associations were observed in a subgroup of conditionally healthy individuals and in the analysis considering prior PA levels.

Most current evidence has focused on investigating whether PA can improve sleep outcomes^10–13^. The influence of sleep characteristics on subsequent engagement in PA has been less studied in the adult population^21–25^, particularly among older individuals. Existing research has often addressed short-term (day-to-day) variations in sleep and PA, or has been limited to individuals with sleep disturbances, mental disorders, or other medical conditions, as summarized in a meta-analysis^26^. In line with our findings, a longitudinal observational study of sleep and PA in 426 older adults revealed that better sleep quality measured at baseline was associated with greater levels of PA measured two years later. Interestingly, initial PA did not predict later sleep quality in this study^24^. Another investigation based on data from the Helsinki Health Study (*n* = 6458, 40–60 years old at baseline) aiming to explore the bidirectional relationship between insomnia symptoms and unhealthy behaviors showed that insomnia symptoms surveyed at baseline in 2000–2002 have been associated with physical inactivity measured in 2007 defined as less than 14 MET-hours per week (OR [95% CI] = 1.27 [1.08, 1.48]). On the contrary, PA at baseline has not been linked to subsequent insomnia symptoms at follow-up in a fully adjusted model (OR [95% CI] = 1.13 [0.98, 1.30])^25^. Compared to these previous studies, our study included a larger number of participants with a broader age range and investigated the relationship between several sleep and PA characteristics.

In line with our findings, the link between sleep duration and PA has been previously reported in population-based studies^21–23^. In a cross-sectional study of 2,179 generally healthy adolescents, sleep duration of fewer than 8 h per day was associated with lower odds of engagement in leisure-time PA and higher odds of excessive TV watching in males but not females^21^. A cross-sectional study of UK Biobank participants (*n* = 82,995; aged 43–79 years) revealed a U-shaped relationship between objectively measured sleep duration and PA, with the highest PA observed among those who slept 6–7 h per night^23^. In addition, several studies suggested lower PA levels in patients with obstructive sleep apnea, the most common SDB disorder characterized by repeated episodes of apneas, hypopneas, and/or respiratory effort-related arousals caused by repetitive collapse of the upper airway during sleep^34,35^. The evidence of the link between symptoms of SDB and engagement in different types of PA in apparently healthy individuals is limited.

Our study has also shown that the link between symptoms of SDB and sedentary behavior was more pronounced in men than in women. In addition, a stronger inverse association between symptoms of SDB and exercise and a stronger direct relationship between short sleep duration and sedentary behavior were found in middle-aged participants compared with older individuals. To our knowledge, no previous population-based study investigated whether the link between sleep and PA characteristics was modified by sex or age. This can be related to the smaller sample size in several previous studies.

The mechanisms underlying the association between sleep characteristics and subsequent engagement in PA likely involve several pathways. Poor sleep has been linked to fatigue, impaired cognitive ability and concentration, an increased risk of stress-related disorders, social withdrawal and loneliness, as well as substance use^14–18^. This may, in turn, reduce the motivation to engage in PA. For example, experimental and observational studies have consistently demonstrated that tiredness and fatigue typically arise from the physiological effects of insufficient sleep, poor sleep quality, SDB, and being awake during circadian periods when the brain is programmed to sleep^17,18^. A longitudinal trajectory analysis based on a cohort of 3,265 adolescents and young adults suggested that worsening of sleep health (e.g., bedtime and trouble sleeping) was associated with an increased likelihood of alcohol and cannabis use over time^14^. An experimental study in 48-hour abstinent cigarette smokers (mean age of 26 years) has shown that sleep deprivation is linked to a higher level of self-reported fatigue, decreased arousal, impaired behavioral inhibition and attention, and higher smoking levels following a night of sleep deprivation compared to a night of normal sleep^15^. In addition, there is compelling evidence that poor sleep may lead to increased caloric intake, unhealthy dietary choices, and, as a consequence, excessive body weight^19^. This may also mediate the relationship between unfavorable sleep characteristics and PA. Moreover, adequate sleep is important for muscle recovery and physical performance^20^.

Our study has several strengths, including a large sample size, assessment of several sleep variables and PA parameters, adjustment for important confounders, such as objectively assessed health status, and inclusion of both women and men. In addition, several sensitivity analyses were conducted given the bidirectional relationship between sleep and PA and the large influence of health status on both exposures and outcomes of interest. Several limitations, however, apply to the present observational study. Since our study included women and men of mainly northern European origin, our findings might be not generalizable to other ethnic groups. This cohort included relatively old participants, and the observed associations may differ in younger populations. As in several other population-based observational studies^21,22,24,25^, sleep duration, sleep disturbance, and SDB symptoms were self-reported. Another limitation is that the follow-up period between sleep characteristics (questionnaire in 2008) and PA measures (2009) was relatively short, and the exact follow-up time was not available for each participant. As a result, some overlap between exposure and outcome periods may have occurred, increasing the risk of reverse causation. However, substantial temporal overlap is unlikely since sleep duration reflected average patterns, and sleep complaints referred to the preceding three months. Information on diagnosed insomnia, SDB, or other clinical sleep-related conditions, as well as related medication use, was not available and therefore could not be accounted for in the analyses. Due to the observational nature of this study, we cannot rule out residual and unmeasured confounding.

In conclusion, short or long sleep duration, sleep disturbance symptoms, and symptoms of SDB were common in this cohort of middle-aged and older participants. A higher prevalence of short and long sleep, as well as sleep disturbance symptoms, was found in women, whereas symptoms of SDB were more common in men. In addition to compelling evidence of the positive effect of PA on sleep, our study has indicated that poor sleep may also contribute to low PA levels and higher levels of sedentary behavior, emphasizing a robust bidirectional relationship between PA and sleep. Good sleep reduces stress and promotes health and recovery, thereby increasing the likelihood of engaging in PA, which also directly affects health and well-being. Therefore, a better understanding of the association between sleep characteristics and PA across age groups is important for researchers and public health policymakers to develop intervention programs that promote good sleep and adequate physical activity.

## Electronic supplementary material

Below is the link to the electronic supplementary material.

Supplementary Material 1

## Data Availability

The datasets analyzed during the current study are not publicly available because due to Swedish laws on personal integrity and health data, as well as the decision by the Ethics Committee, we are not allowed to make any data, including health variables, open to the public, even if made anonymous. The data that support the findings of this study are available upon application to the Swedish Infrastructure for Medical Population-based Life-course Environmental Research (SIMPLER).
