# Supplementary material for: Poor sleep is associated with lower physical activity in a population-based cohort of middle-aged and older adults
Source: Sci Rep. 2025 Jul 17;15:26012. doi: 10.1038/s41598-025-10991-2 (PMC12271448; doi:10.1038/s41598-025-10991-2)
Supplement: Supplementary file 1 — Supplementary Material 1 [file 41598_2025_10991_MOESM1_ESM.pdf]

Supplementary Material

Poor sleep is associated with lower physical activity in a population-based cohort of middle-aged and older adults

Susanna C Larsson, Emma Hållström, Karl Michaëlsson, and Olga E Titova

Content	***Page***
**Supplementary information.** Methods	2
**Supplementary Table 1.** Associations between sleep variables (2008) and engagement in physical activity (2009) in participants with Charlson's comorbidity index =0, n=**36,875**	3-4
**Supplementary Table 2.** Associations between sleep variables (2008) and engagement in physical activity (2009), additionally adjusted for physical activity surveyed in 1997	5-6

Supplementary information. Methods

SIMPLER, cohort description

Participants born between 1914 and 1952 were invited to participate in two longitudinal SIMPLER cohorts, the Swedish Mammography Cohort (SMC) and the Cohort of Swedish Men (COSM). The SMC was established between 1987 and 1990, when all women living in Västmanland and Uppsala counties (Sweden) and born between 1914 and 1948 received a questionnaire on dietary habits and other characteristics (90,303 women invited, 74% response rate). The COSM was established in 1997 and included men living in Västmanland and Örebro counties (Sweden), born between 1918 and 1952 (100,303 men invited, 49% response rate). In 1997, the 56,030 participants in SMC who were still alive and resided in the study area received an expanded diet and lifestyle questionnaire similar to one sent to COSM participants (70% response rate).[1,2] In 2008, a new *Health questionnaire* was sent to the participants in SMC and COSM who were alive (response rate: 63% in SMC and 78% in COSM). In 2009, SMC and COSM participants who completed the health questionnaire in 2008 received an updated *Diet and lifestyle questionnaire* (response rate: 84% in SMC and 90% in COSM).[2] The questionnaires were identical besides specific questions relevant for women or men (e.g., related to the age of menopause or problems with urination). According to previous research based on these cohorts, SMC and COSM are representative of the Swedish population in terms of age distribution, educational level, and prevalence of overweight and obesity. [3]

References:

1. Titova OE, Baron JA, Michaëlsson K, Larsson SC. Anger frequency and risk of cardiovascular morbidity and mortality. Eur Heart J Open. 2022 Aug 6;2(4):oeac050.

2. Harris H, Håkansson N, Olofsson C, Stackelberg O, Julin B, Åkesson A, Wolk A. The Swedish mammography cohort and the cohort of Swedish men: study design and characteristics of 2 population-based longitudinal cohorts. OA Epidemiol 2013;1:16.

3. Stackelberg O, Bjorck M, Larsson SC, Orsini N, Wolk A. Sex differences in the association between smoking and abdominal aortic aneurysm. Br J Surg. Sep 2014;101(10):1230-7. doi:10.1002/bjs.9526

Supplementary Table 1. Associations between sleep variables (2008) and engagement in physical activity (2009) in participants with Charlson's comorbidity index =0, n=**36,875**

	**Model A*** OR (95% CI)	**Model B**** OR ((95% CI)
**Walking / cycling > 40min/day**		
**Sleep duration** (h/night)		
<7	**0.95 (0.91-0.99)**	0.99 (0.94-1.04)
7 - <9	Ref	Ref
≥ 9	**0.84 (0.76-0.92)**	**0.82 (0.74-0.90)**
**Sleep disturbance** #		
No	Ref	Ref
Yes	**0.87 (0.83-0.90)**	**0.86 (0.83-0.90)**
**Symptoms of SDB** #		
No	Ref	Ref
Yes	**0.74 (0.70-0.78)**	**0.81 (0.77-0.85)**
**Exercise ≥ 2 hour/week**		
**Sleep duration** (h/night)		
<7	0.95 (0.89-1.01)	0.98 (0.92-1.05)
7 - <9	Ref	Ref
≥ 9	**0.77 (0.67-0.89)**	**0.77 (0.67-0.88)**
**Sleep disturbance** #		
No	Ref	Ref
Yes	0.96 (0.90-1.02)	0.95 (0.90-1.00)
**Symptoms of SDB** #		
No	Ref	Ref

Yes	**0.86 (0.80-0.92)**	**0.91 (0.85-0.98)**
**Reading/watching TV > 4h/day**		
**Sleep duration** (h/night)		
<7	**1.27 (1.16-1.39)**	**1.27 (1.16-1.39)**
7 - <9	Ref	Ref
≥ 9	1.14 (0.96-1.35)	0.99 (0.83-1.17)
**Sleep disturbance** #		
No	Ref	Ref
Yes	**1.33 (1.22-1.45)**	**1.24 (1.14-1.36)**
**Symptoms of SDB** #		
No	Ref	Ref
Yes	**1.25 (1.13-1.38)**	**1.14 (1.03-1.26)**

Abbreviations: CI, confidence interval; OR, odds ratio; Ref, reference group for the analysis.
* Model A was adjusted for age, sex, and education based on binary logistic regression.
** Model B was further adjusted for employment status, cohabiting status, cigarette smoking, alcohol consumption, body mass index, BMI, and history of depression.
# Participants reported that at least one symptom (see methods for description) occurred often, most often, or always. Bold values = P-values < 0.05.

Supplementary Table 2. Associations between sleep variables (2008) and engagement in physical activity (2009) additionally adjusted for physical activity surveyed in 1997

	**Model A*** OR (95% CI)	**Model B**** OR ((95% CI)
**Walking / cycling > 40min/day,** n=44,187		
**Sleep duration** (h/night)		
<7	**0.92 (0.88-0.97)**	0.97 (0.93-1.02)
7 - <9	Ref	Ref
≥ 9	**0.78 (0.72-0.85)**	**0.79 (0.72-0.86)**
**Sleep disturbance** #		
No	Ref	Ref
Yes	**0.85 (0.82-0.89)**	**0.86 (0.83-0.90)**
**Symptoms of SDB** #		
No	Ref	Ref
Yes	**0.76 (0.72-0.80)**	**0.83 (0.78-0.87)**
**Exercise ≥ 2 hour/week,** n=43,601		
**Sleep duration** (h/night)		
<7	0.97 (0.91-1.02)	1.00 (0.94-1.06)
7 - <9	Ref	Ref
≥ 9	**0.84 (0.74-0.94)**	**0.84 (0.74-0.94)**
**Sleep disturbance** #		
No	Ref	Ref
Yes	**0.94 (0.89-0.99)**	**0.94 (0.89-0.99)**
**Symptoms of SDB** #		
No	Ref	Ref
Yes	**0.91 (0.85-0.97)**	0.95 (0.89-1.02)

**Reading/watching TV > 4h/day,** n=44,752		
**Sleep duration** (h/night)		
<7	**1.18 (1.09-1.28)**	**1.17 (1.08-1.27)**
7 - <9	Ref	Ref
≥ *9*	1.09 (0.95-1.26)	0.96 (0.83-1.11)
**Sleep disturbance** #		
No	Ref	Ref
Yes	**1.26 (1.17-1.36)**	**1.18 (1.09-1.27)**
**Symptoms of SDB** #		
No	Ref	Ref
Yes	**1.24 (1.14-1.35)**	**1.14 (1.05-1.25)**

Abbreviations: CI, confidence interval; OR, odds ratio; Ref, reference group for the analysis.
* Model A was adjusted for age, sex, respective PA measured in 1997, and education based on binary logistic regression. Missing values for PA in 1997 were not imputed, resulting in differences in sample size.
** Model B was further adjusted for employment status, cohabiting status, cigarette smoking, alcohol consumption, body mass index, BMI, Charlson's Comorbidity Index, and history of depression.
# Participants reported that at least one symptom (see methods for description) occurred often, most often, or always. Bold values = P-values < 0.05.
